# Conformational Flexibility in Capsids Encoded by the *Caliciviridae*

**DOI:** 10.3390/v16121835

**Published:** 2024-11-26

**Authors:** Charlotte B. Lewis, Lee Sherry, Michaela J. Conley, Masaaki Nakashima, Shirin Akbar, Nithya Govindan, Margaret J. Hosie, David Bhella

**Affiliations:** MRC—University of Glasgow Centre for Virus Research, Garscube Campus, 464 Bearsden Road, Glasgow G61 1QH, UK; c.lewis.2@research.gla.ac.uk (C.B.L.); lee.sherry@glasgow.ac.uk (L.S.); michaela.conley@sartorius.com (M.J.C.); masaaki.nakashima@shionogi.co.jp (M.N.); shirin.akbar@icm.uu.se (S.A.); margaret.hosie@glasgow.ac.uk (M.J.H.)

**Keywords:** calicivirus, capsid, P-domain, structure, cryo-EM

## Abstract

Caliciviruses are a diverse group of non-enveloped, positive-sense RNA viruses with a wide range of hosts and transmission routes. Norovirus is the most well-known member of the *Caliciviridae*; the acute gastroenteritis caused by human norovirus (HuNoV), for example, frequently results in closures of hospital wards and schools during the winter months. One area of calicivirus biology that has gained increasing attention over the past decade is the conformational flexibility exhibited by the protruding (P) domains of the major capsid protein VP1. This was observed in structure analyses of capsids encoded by many species and is often a consequence of environmental cues such as metal ions, changes to pH, or receptor/co-factor engagement. This review summarises the current understanding of P-domain flexibility, discussing the role this region plays in caliciviral infection and immune evasion, and highlighting potential avenues for further investigation.

## 1. Introduction

The *Caliciviridae* family comprises positive-sense RNA viruses of medical and veterinary significance, having been found to infect a wide variety of vertebrate species, including birds, reptiles, as well as humans and several other mammal species [1]. As determined by the ICTV, the *Caliciviridae* currently contains 11 established genera, and 13 recognised species, although novel caliciviruses are regularly discovered through improved sequencing and sampling technologies [1,2].

Caliciviruses are encoded through linear, single-stranded genomes that vary between 7.3 and 8.5 kb in length. The overall genome structure begins with a well-conserved 5′ untranslated region (UTR) that is covalently linked to VPg (viral protein genome-linked), followed by a polycistronic open-reading frame (ORF) and 3′ UTR culminating in a poly-A tail [3,4]. Despite sharing these features, the genome organisation differs between the different genera, with Bavo-, Lago-, Mino-, Naco-, Nebo-, Salo-, Valo-, and Sapoviruses consisting of two ORFs, while other caliciviruses (Reco-, Vesi-, and Norovirus) containing three ORFs. ORF 1 encodes the non-structural proteins (NS1-NS7), which are translated as a large polyprotein precursor that is co- and post-translationally processed into mature viral proteins [3,4]. 

The translation of the major structural protein VP1 differs slightly between the ‘two-ORF’ and ‘three-ORF’ genera. In those viruses having two ORFs, VP1 is located at the C-terminal end of ORF1 and therefore can be produced from proteolytic cleavage of the ORF1-encoded polyprotein. However, the vast majority of VP1 is produced from a subgenomic RNA (sgRNA) at later stages of infection prior to virion assembly and genome encapsidation [5,6]. In the ‘three-ORF’ model, VP1 is encoded by ORF2, and although some level of ribosomal read-through leads to VP1 translation from the genomic RNA, nearly all VP1 is translated from the sgRNA during infection [7]. In both models, the last ORF encodes the minor capsid protein, VP2, which has been shown to be essential for infectivity [8,9]. 

Caliciviruses are associated with severe diseases in both humans and animals. From a veterinary perspective, there are several important caliciviruses. For example, feline calicivirus (FCV), the causative agent of “cat flu”, usually presenting as a self-limiting respiratory disease. Recently, FCV has also been responsible for outbreaks of virulent and often lethal infections in cats, associated with emerging virulent systemic strains of FCV (VS-FCV) that have been reported worldwide [10]. VS-FCV infection is characterised by systemic vasculitis and severe epithelial necrosis that leads to oedema, ulceration of the skin, jaundice, and pneumonia. Interestingly, there are striking clinical similarities between the disease observed in cats infected with VS-FCV and the presentation of diseases caused by European brown hare syndrome virus (EBHSV) and rabbit haemorrhagic disease virus (RHDV), which are often fatal [10,11,12]. Another virus of veterinary importance, vesicular exanthema of swine virus (VESV), infects pigs and marine mammals causing acute disease, characterized by the formation of vesicles on the oral mucosa in marine mammals, as well as the snout and feet in swine, with these clinical signs accompanied by high fever. VESV is an important differential diagnosis in the veterinary community as clinically, it is hard to distinguish from foot-and-mouth disease [13]. 

The major human caliciviruses are the noroviruses (NoV) and the sapoviruses (SaV), causing acute gastroenteritis and significantly impacting human health. Human NoV (HuNoV), commonly known as the ‘winter vomiting bug,’ causes approximately 3.7 million cases of acute gastroenteritis in the UK annually [14]. Whereas globally, HuNoV and SaV are responsible for approximately 212,000 deaths each year and are a leading cause of infant mortality in low- and middle-income countries. The very young, the elderly, and immunocompromised individuals are at greatest risk from HuNoV infections. While this causes typically self-limiting illness in healthy people, the societal and economic burden of HuNoV infection should not be underestimated due to its capacity to cause large-scale outbreaks, which frequently occur in schools, hospitals, and care homes [15]. Despite the clear need for vaccines and antivirals, the limited cultivability of HuNoV in cell culture systems has hindered the development of clinically approved therapeutics. Although recently, organoid and in vivo models for HuNoV infection have been reported, these studies are still in their infancy and most of our current understanding of HuNoV has been inferred from other cultivatable members of the *Caliciviridae*, such as murine norovirus (MNV), FCV, and Tulane virus (TuV). These viruses have been widely used in structural, infectivity, environmental survivability, and disinfection studies due to their genetic and structural similarities to HuNoV [16,17,18]. 

Despite considerable efforts, attempts to produce effective vaccines for norovirus have proven challenging, as not only do noroviruses display considerable genetic diversity, which leads to the generation of new epidemic strains every 2–4 years [19,20,21], the viral capsids are dynamic, likely contributing to their ability to evade neutralisation. Conformational flexibility of the capsid spikes formed by the protruding domains of VP1 has been observed in several different caliciviruses, initially confounding efforts to describe capsid structure and characterise viral entry pathways. Here, we provide an overview of the environmental cues and receptor interactions that may prompt these conformational changes across multiple caliciviruses. We provide new data to show that conformational flexibility in TuV capsids, for which we have yet to identify an environmental cue, varies between preparations, potentially frustrating efforts to study entry. Additionally, we discuss how conformational flexibility of P-domains is essential for the assembly of the calicivirus portal, formed of the minor capsid protein VP2, which in FCV has been shown to mediate genome release into the cytoplasm to initiate infection [22,23]. 

## 2. Materials and Methods

### 2.1. Virus Culture

Tulane virus (TuV) was propagated in LLC-MK2 cells. Thirteen roller bottles with confluent monolayers of LLC-MK2 cells were infected with TuV at an MOI of 0.2 for 24–72 h, after which time there was extensive cytopathic effect, and the majority of cells had died. Culture media was decanted, and the cell debris was pelleted at 5000 rpm (TX-400, Thermo Fisher Scientific, Waltham, MA, USA) and subjected to freeze thawing before being resuspended in 5 mL of PBS. Both resuspended pellet and supernatant fractions were then ultracentrifuged at 28,000 rpm (SureSpin630, Thermo Fisher Scientific). The resulting pellets were then loaded onto 31–42% CsCl gradients and ultracentrifuged at 28,000 rpm for 18 h at 12 °C. The virus-containing bands were pooled and pelleted at 20,000 rpm for 2 h and resuspended in 50 µL PBS. 

### 2.2. Cryo-EM

Cryo-EM grids of TuV were prepared using a Leica GP2 vitrification robot. Briefly, 4.5 µL of virus suspension was loaded onto freshly glow-discharged quantifoil holey carbon support films (R2/2 300 mesh), blotted for 2 s, and plunged into a bath of liquid nitrogen-cooled liquid ethane. 

Grids were imaged in a JEOL CryoARM 300 at 60 k× magnification with micrograph movies recorded on either a Direct Electron 64 detector at a frame rate of 25 frames per second (resting state preparation), or Direct Electron Apollo detector with movies written at 20 frames per second (raised state C/C-dimer) and a ~60 e/Å2 total dose. In both cases, 2-second exposures were captured. DE64 images were recorded in integrating mode, while Apollo images were recorded in super-resolution counting mode. 

### 2.3. Image Processing and Model Building 

A total of 3475 micrograph movies of TuV recorded on the DE64 (resting state), and 7746 micrograph movies recorded on the DE Apollo (raised state), were processed using Relion 3.1 [24,25] to calculate 3D reconstructions with icosahedral symmetry imposed as previously described [26]. An initial model for the resting state map was assembled using ModelAngelo [27] edited using Coot [28] and refined using Phenix [29] and Isolde [30]. 

To model the conformational changes at the C/C dimer, focussed classification was used to calculate a reconstruction with C1 symmetry as previously described [22,31]. The class that showed the clearest density for the tilted C/C P-dimer was then subjected to local sharpening using DeepEMhancer [32]. The P- and S- dimer domains of a single C/C dimer model were separately docked as rigid bodies at the site of the tilted capsid spike using UCSF Chimera [33] and manually edited in Coot to rebuild the linker region. The resulting coordinates were then refined using Phenix. Maps and models were visualised using UCSF ChimeraX [34]. 

## 3. Capsid Structure of the *Caliciviridae*

**Figure 1:**
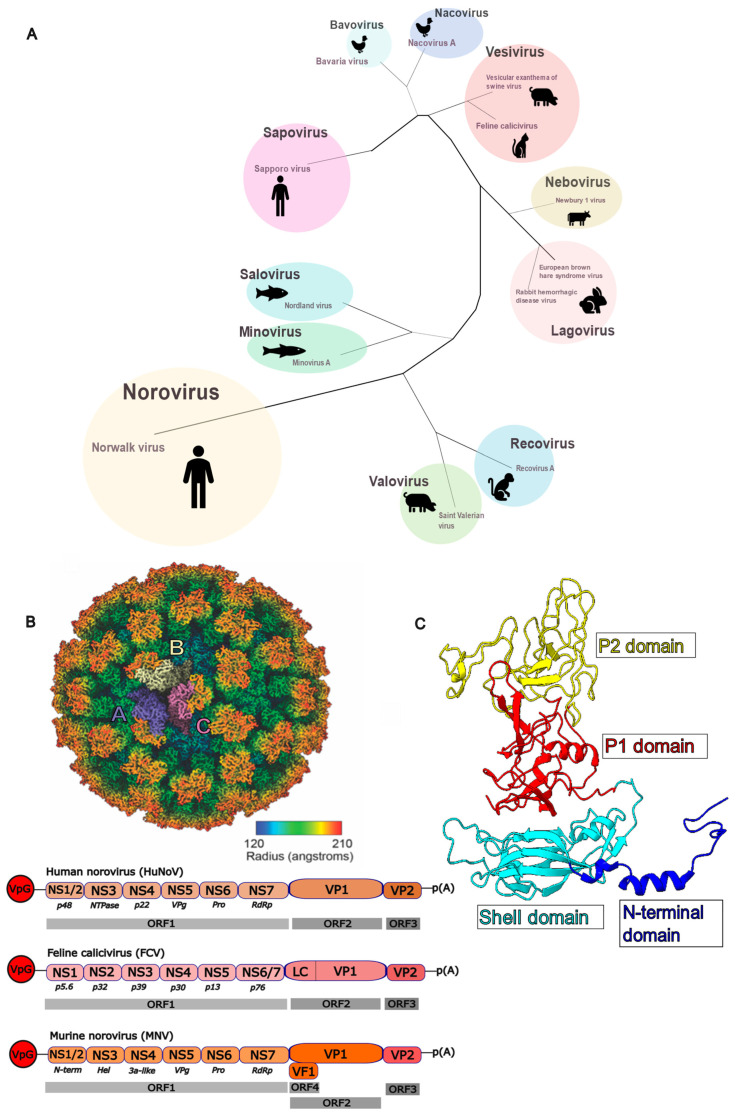
(**A**) Simplified phylogenetic illustration of the *Caliciviridae* adapted from the phylogenetic tree presented in the ICTV Virus Taxonomy Profile [1]. The tree is based on amino acid sequences of the major capsid protein VP1. The tree illustrates the 11 recognised genera (*Sapovirus*, *Bavovirus*, *Nacovirus*, *Vesivirus*, *Nebovirus*, *Lagovirus*, *Recovirus*, *Valovirus*, *Norovirus*, *Minovirus*, and *Salovirus*) and 13 recognised species of the *Caliciviridae* alongside illustrations of representative hosts (non-exhaustive). (**B**) Cryo-EM structure of FCV [22], coloured according to the radius (key) with three protomers coloured to identify the positions of the A (purple), B (cream), and C (magenta) quasiequivalent positions of VP1. Depicted below the cryo-EM structure are schematic representations of the HuNoV, FCV, and MNV genomes. Adapted from [4]. The 5′ end of calicivirus genomes is covalently linked to viral protein genome-linked (VPg) proteins and a poly(A) tail of variable length at the 3′ end (depicted as p(A)). ORF1 encodes a viral polyprotein that is cleaved by virally encoded proteases into multiple non-structural proteins. In all three viruses, ORF2 and ORF3 encode the major capsid protein VP1 and minor capsid protein VP2, respectively; both are translated from a bicistronic subgenomic RNA (sgRNA) at later stages of infection. FCV ORF2 also encodes a leader of the capsid (LC) protein. ORF4, found only in MNV, is a fourth overlapping reading frame within ORF2 that encodes virulence factor 1 (VF1). NTPase = Nucleoside triphosphatase, Pro = Protease, RdRp = RNA-dependent RNA polymerase, N-term = N-terminal protein, Hel = Helicase. (**C**) Ribbon diagram illustrating the N-terminal domain (blue), shell domain (cyan), P1 (red), and P2 (yellow) domains of the major capsid protein VP1 (PDB 6GSH [22]).

The main component of the calicivirus capsid is the ~60 kDa major capsid protein VP1, which typically assembles into a T = 3 icosahedral shell [35,36]. Thus, each capsid consists of 180 copies of VP1, arranged in three quasi-equivalent conformations designated A, B, and C. The A/B subunits form dimers around the five-fold axis, while C/C subunit dimers are located at the two-fold axis (Figure 1B [37,38]). 

Each VP1 consists of three distinct regions: the N-terminal domain (N); the shell (S)-domain, which surrounds the viral genome; and the protruding (P)-domain located on the outer surface of the capsid, which forms the characteristic arch-like protrusions observed in the structures of many caliciviruses. Each P-domain is divided into sub-domains, P1 and P2, with P1 as the ‘base’ of the P-domain and connected to the S-domain through an ~8–12 aa flexible hinge. The P2-domain is on the outermost part of the capsid (Figure 1C). 

**Figure 2:**
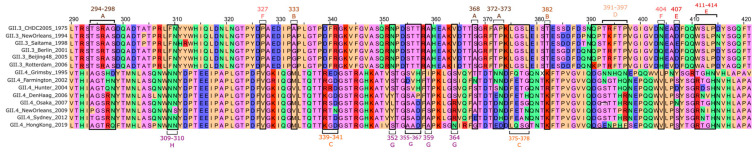
Amino acid sequence alignment of highly variable antigenic regions A−H from selected HuNoV GII.3 and GII.4 human norovirus isolates, located on the major capsid protein VP1. Residue locations (from Tohma et al. and Lo et al.) are illustrated by boxes and brackets. Antigenic region A: residues 294, 295, 296, 297, 298, 368, 372, 373. B: 333, 382. C: 339, 340, 341, 375, 376, 377, 378. D: 393, 394, 395, 396, 397. E: 407, 411, 412, 413, 414. F; 327, 404. G: 352, 355, 356, 357, 359, 364. H: 309, 310 [51,54]. Residues are coloured according to biochemical properties. Pink = non-polar (Gly, Ala, Ser, Thr); orange = hydrophobic (Cys, Val, Ile, Leu, Pro, Phe, Tyr, Met, Trp); green = polar (Asn, Gln, His); blue = negatively charged (Asp, Glu); Red = positively charged (Lys, Arg). ORF2 sequences were downloaded from GenBank [55] and aligned and visualised in Jalview 2.11.4.0 [56]. GenBank sequence identifiers and protein IDs can be found in Supplementary Table S1.

The P2-domain plays a significant role in receptor attachment and contains major immunodominant epitopes. This domain exhibits a considerable amount of amino acid sequence variability compared to the relatively conserved P1 [39,40,41], with antigenic drift driving the emergence of immune escape variants [42,43] (Figure 2). This antigenic drift has been most extensively studied in the context of HuNoV. For example, residue alterations in GII.4 antigenic sites have been associated with altered antigenicity and potential immune escape of pandemic variants. Antigenic region A (comprising residues 294, 296, 297, 298, 368, 372, and 373) undergoes a high rate of mutation across GII.4 strains, leading to distinct antigenic profiles [44]. The co-evolution of residues 297 and 372 of region A has been associated with altered antigenicity of the Sydney 2012 GII.4 pandemic strain [45,46], and mutations in residues 294–296 of region A as well as residues 393–395 (region D) appear to impair monoclonal antibody (mAb) recognition [47]. Additionally, evolution of sites A and D has been associated with the antigenic escape of GII.4 Sydney 2012, in comparison with earlier variants such as GII.4 New Orleans 2009 [46]. Antigenic site A appeared to be primarily immunodominant in earlier variants such as GII.4 Farmington Hills (2002) before being superseded by antigenic site G in later variants such as GII.4 Sydney (2012), highlighting the role of these regions in generating immune escape variants [48].

Region B is buried in the dimer interface as opposed to the P2-domain surface. It has been suggested that variation at these residues might alter the exposure and/or physiochemical properties of surface-exposed residues in other epitopes. In a similar vein, antibody binding to antigenic regions E and F appear to be temperature-sensitive, suggesting that these sites are only transiently exposed rather than displayed on the capsid surface [49,50]. Variations at the recently identified antigenic site C as well as the more conserved site G appear to correlate with the emergence of new GII.4 variants [51], with a moderate impact on antigenicity in the case of site C [48]. Site C is located proximal to the histo-blood group antigen (HBGA) binding pocket, suggesting amino acid changes in this region could influence HBGA binding; a mAb targeting residues 340 and 376 of this region in GII.4-MD2004 HuNoV inhibited HBGA interactions in a strain-specific manner [52]. Variation in residues of antigenic site D, specifically 393 to 395, have also been associated with modulation of HBGA affinity, as well as affecting antibody binding [43,53].

The role of the ~12 kDa minor capsid subunit VP2 was, until recently, less well-characterised. It has been previously shown to be essential for the development of infectious particles [9] with VLP studies indicating that VP2 interacts with VP1 to facilitate virion stability [57]. We showed that following receptor engagement by FCV, 12 VP2 monomers come together to form a portal at a unique three-fold axis. Portal assembly is accompanied by substantial capsid rearrangements including rotation of surrounding P-dimers towards the portal vertex to buttress the assembly [22]. Our hypothesis that the VP2 portal mediates genome delivery following endocytosis was recently supported by a study that showed FCV VP2 was both able to form a pore and facilitate viral genome release in a liposome model [23]. 

## 4. P-Domain Conformation Is Altered by Environmental Cues

**Figure 3:**
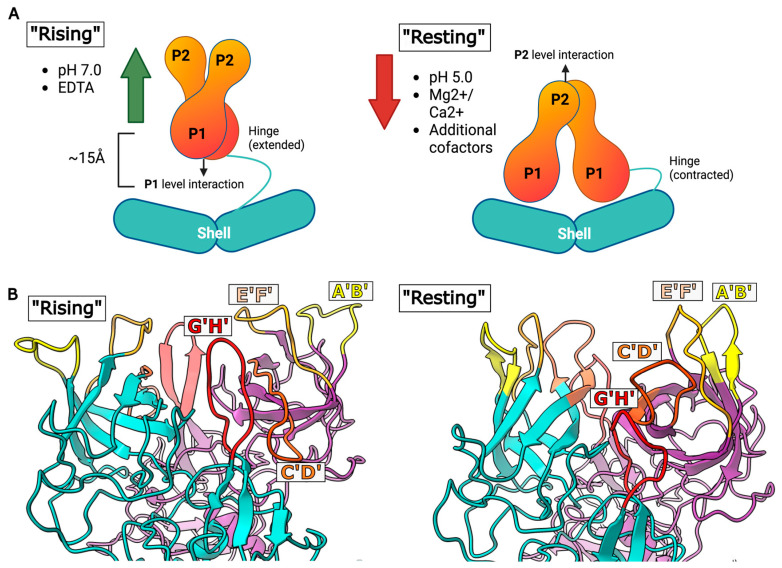
(**A**) Simplified representation of P-domain rotation and collapse. Image created in Biorender. (**B**) Cartoon diagram of suggested loop movements in the resting and rising P-domain positions in the P2 regions of an A/B dimer. A dimer is coloured cyan, B dimer is coloured purple. G’H’ loop is coloured red, C’D’ loop is coloured orange, E’F’ loop is coloured light orange, and A’B’ loop is coloured yellow. Note the significant upwards movement of the C’D’ loop in the rising P-domain position. Adapted from [66]. PDB of resting position: 7N6Y [67]. PDB of rising position: 7L5J [68].

Calicivirus P-domains display two major conformations, a ‘rising’ conformation and a ‘resting’ conformation (Figure 3). The ‘resting’ P-domain conformation, in which the flexible hinge is contracted and P-domains rest upon the shell, was previously reported in HuNoV strain GI.1 [36], GI.4 [58], San Miguel sea lion virus [37], and sapovirus [59]. 

Conversely, P-domains in MNV were identified in a ‘rising’ conformation wherein the A-B and C-C dimers lifted off the capsid shell by approximately 16 Å and rotated clockwise by approximately 40° [60]. This was suggested to be similar to the capsid maturation observed in reovirus [61] and a strategy for facilitating receptor engagement. A similar phenomenon was observed for HuNoV strain GII.10 [62], as well as FCV upon receptor binding [63] and RHDV, although in this instance the P-domains were raised but had not undergone rotation [64]. 

Importantly, it was later found by Song et al. that P-domains of HuNoV GII.3 VLPs could reversibly transition between both conformations [65], demonstrating that the P-domains of an individual virus could adopt both the resting and rising forms. This important discovery highlighted that P-domains on each viral particle were able to move interchangeably between resting and rising states, rather than remaining irreversibly ‘fixed’ in one conformation. Following this observation, several studies have looked to define the environmental cues that cause these transitions. Here, we summarise the impact of metal ions, pH, and additional co-factors on P-domain conformation, as well as the potential roles of capsid flexibility in calicivirus infection. 

## 5. Metal Ions

Metal ions play a key role in a variety of cellular and subcellular processes, from modulating enzyme activity, signal transduction, and structural co-ordination of host proteins and receptors. The physiological concentrations of these ions differ between physiological systems and cellular compartments; therefore the homeostatic control of metal ions is essential. At the viral level, metal ion concentration was implicated as a possible factor in HuNoV receptor interactions [69,70]. Ion flux during endosome maturation suggests ion concentration as a potential environmental cue required for efficient viral entry. Song et al. reported that treating MNV in which P-domains were in the resting conformation, with the chelating agent EDTA at pH > 7, triggered a transition to the rising state. P-domains were shown to rise off the shell and rotate clockwise by approximately 70°. Conversely, incubation with DMEM caused P-domains to fall back into the resting state within 2–8 h [65]. This was suggested to be due to the presence of metal ions in DMEM, although it was not known which specific metals were involved. 

Following on from this, it was found that incubation with solutions of 1mM CaCl_2_ or MgCl_2_ was sufficient to cause the contraction of the P-domains into the ‘resting’ conformation. Dialysis against EDTA resulted in P-domains lifting off the shell and exhibiting a high level of disorder, providing strong evidence that divalent cations such as Mg^2+^ and Ca^2+^ are responsible for the transition to a ‘resting’ state [66]. While the structural basis for this movement has not yet been fully elucidated, Sherman et al. propose a mechanism by which significant structural changes occur in four flexible loops at the tip of the MNV P2-domain in response to environmental cues. In this model, the metal ions are suggested to bind in a negatively charged pocket at the base of the G’H’ loop; this causes the G’H’ loop to distort, which triggers P-domain collapse by shifting the usually downwards-facing C’D’ loop into an upwards position. This pushes the A’B’ and E’F’ loops together. These sequential changes cause the rotation and subsequent resting of the P-domains onto the MNV shell. Although the P-domain region is stabilised by the binding of divalent ions, these loops remain mobile, with the C’D’ and D’E’ loops remaining flexible [66]. 

The presence or absence of metal ions has also been shown to influence HuNoV capsid dynamics. Upon treating HuNoV GII.4 VLPs with EDTA, Hu et al. found that capsid diameter increased by approximately 4 nm at pH 6.0 and 13 nm at pH 8.0, suggesting that metal chelation had caused P-domains to transition to the ‘rising’ conformation [69]. Cryo-EM density maps revealed evidence of a conserved Histidine at position 460 with affinity for metal ions at a central position between the A/B and C/C dimers. The location of this residue, as well as its conservation in both A/B and C/C dimers, suggests that metal ion binding in this site may stabilise HuNoV P-domains in the resting position. In the HuNoV GII.4 VLP crystal structure, a cadmium (Cd^2+^) ion appeared to be bound at this residue; this was likely caused by the VLP crystals being produced in a solution containing cadmium chloride (CdCl_2_) but highlights the potential for metal ions to bind at this site. Alongside this, an unidentified metal ion density was present in the cryo-EM structure [69]. A Cys452-Cys452 disulfide bond in the same central position in-between A/B and C/C subunits was identified in a cell-culture-adapted Tulane virus, suggesting that stabilisation of P-dimers at a central point is a conserved feature of the *Caliciviridae* [71]. Similarly to His, Cys also has a putative role in coordinating metal ions [72]. However, to our knowledge, the location of metal-binding sites in TuV have not yet been described. 

Notably, residues involved in metal ion binding in the GII.4 HuNoV genotype, which has been associated with severe gastroenteritis symptoms [73] and epidemic-level outbreaks [74,75], contain a H→Y substitution at position 460 and an L→Q substitution at position 459. These do not appear to be present in less virulent strains, where the sequence is highly conserved [69]. While the impact of this is unknown, it was suggested that this may increase affinity for metal ions in the environment. Alternatively, as this site is close to the hinge region, GII.4 strains may exhibit an altered preference for the resting or rising conformation or require a lower concentration of metal ions in the environment to undergo conformational transitions compared to strains without these substitutions. 

Similarly, while the effect of metal ions on the FCV capsid has not been directly studied, a putative metal ion binding site was identified in the P2-domain of the FCV vaccine strain F9 [22]. Conversely, no evidence for metal binding could be found in the VS strain FCV-5. This may suggest an altered affinity for metal ions in VS strains, although this has not yet been fully explored. Nevertheless, alanine mutagenesis of amino acid residues near the metal coordinating sphere of this region in FCV-5 VP1 resulted in particles that could still bind the fJAM-A receptor but were noninfectious [76], potentially due to an inability of the virus to undergo a conformational change that would allow for post-binding steps to take place. It should be noted, however, that FCV is a respiratory pathogen and may not be as responsive to metal ions as enteric caliciviruses such as MNV and HuNoV, although it has been known to acquire enteric tropism [77]. 

For enteric pathogens such as MNV and HuNoV, the responsiveness of P-domains to environmental cations may allow for greater survival in the harsh gastrointestinal environment, such as by acting as a protective mechanism to prevent denaturation or cleavage of the viral capsid by the many digestive enzymes present. Williams et al. suggest that MNV would remain contracted in the metal-rich environment of the stomach and duodenum [67]. This may increase the probability of successful receptor binding as collapsing the P-domain onto the shell increases the available space between the C/C and A/B dimers, which allows for greater accessibility of the CD300lf binding site [78,79]. Then, after MNV enters the colon, where most materials are absorbed, the P-domains may then transition to the raised state [80]. This mechanism may be similar for HuNoV, as the human stomach is known to contain a high concentration of metal ions. The absorption of these cations in the more alkaline intestinal tract may prompt HuNoV P-domains back into the ‘rising’ position [81]. 

The lifting of the P-domains off the shell exposes multiple antigenic sites that are otherwise occluded when the P-domains are contracted [69,82]. It was suggested that this conformational switch also acts as a form of antigenic escape, in which the epitopes presented in the colon are significantly different from those presented in the small intestine. Additionally, the flexible hinge region connecting the shell and P-domains appears to be sensitive to proteases, which are found in high abundance along the digestive tract. The rising conformation transiently exposes the hinge region to protease activity, possibly resulting in its cleavage. This would cause the shedding of some soluble P-domains from the shell, which might act as an alternative target for the immune system–in essence, an immunological ‘distraction’. While this may be fatal for some viruses, due to P-domains being essential for host cell entry, the immunological decoy of the cleaved P-domains may be ‘worth’ the death of some of the population. Additionally, the minimum number of P-domains required for entry is unknown; it may be that a virus may survive the cleaving of a significant number of P-domains and still be able to complete its life cycle. 

TuV may have developed a similar mechanism, also being an enteric pathogen that has a fecal–oral transmission route and is shed in stools [83]; however, to our knowledge, no studies exist on the responsiveness of the TuV capsid to metal ions. Additionally, it should be noted that the minimum concentration of cations required to cause P-domain collapse is currently unknown. Clarifying this would allow for more specific identification of where conformational changes are likely to occur, and if there are genotype-specific differences with regards to the minimum concentration of metal ions required to cause conformational transitions. 

## 6. pH

Although the presence of metal ions has been shown to be sufficient to alter capsid conformation [66], the P-domains of some caliciviruses have also been demonstrated to display differing sensitivities to changes in environmental pH. This is most well-studied using MNV; MNV-S7 required a pH of 8.0 in an EDTA (i.e., metal-chelating) solution for P-domains to transition back to the rising state, in contrast to MNV-1, which required a pH of 7.0 in the same conditions [65]. Additionally, it appears that treatment of MNV strains MNV-1 and WU23 with acidic (pH 5.0) solutions alone is sufficient to cause P-domain collapse [65]. Interestingly, this effect did not extend to MNV strain CR6, which required treatment with the bile acid glycochenodeoxycholic acid (GCDCA) as well as a pH 5.0 solution to cause full contraction. It should be noted that P-domain collapse in MNV increases the available space between the C/C dimer and two A/B dimers, which allows for greater accessibility of the CD300lf binding site. The inability of CR6 to fully transition to the resting state without the synergistic effect of multiple environmental cues may in part explain its lower affinity for the CD300lf receptor than MNV-1, and therefore its reduced infectivity compared to more virulent MNV strains. These findings were supported by Helm et al., who identified that infectivity of MNV-1 and WU23 was enhanced at pH 6.7 compared to pH 7.6, whereas CR6 only exhibited a similar phenotype at the more acidic pH of 5.6 [84]. The reduced responsiveness of CR6 to environmental cues correlated with milder gastrointestinal symptoms in vivo; mice infected with wild-type CR6 exhibited less severe diarrhoea than mice infected with MNV-1 and WU23. As a more virulent strain, MNV-1 may be more responsive to slight changes in pH or exhibit an altered affinity for the CD300lf receptor. The attenuated nature of CR6 in vivo has been noted previously [85] and agrees with Helm et al. that the P2-domain is the main determinant of attenuation. Amino acid K296 in the VP1 P-domains has previously been implicated as a virulence determinant; MNV isolates with this residue, rather than the more common E296, appeared to cause more severe disease in IFN-deficient adult mice [86]. However, this effect could not be recapitulated in immunocompetent neonatal mice, suggesting this phenotypic difference may be due to altered P-domain dynamics between strains. It could be, conversely, that residue changes in VP2 alter these dynamics, but this has not been structurally investigated. 

The effect of environmental changes in pH are less well-studied in HuNoV and TuV. The capsid diameter of HuNoV GII.4 VLPs demonstrated a slight but non-significant expansion from approximately ~43 nm to ~47 nm when incubated in pH 8.0 and 6.0 solutions, respectively [69]. Additionally, biolayer inferometry binding assays between VLPs and NORO-320, a monoclonal antibody which binds to an area on the P-domain occluded in the ‘resting’ position [87], showed no significant difference in binding between pH 8.0 or pH 6.0 conditions. Notable expansions in capsid diameter and NORO-320 binding were only observed once metals were chelated using EDTA, suggesting that low pH is not sufficient to cause capsid transitions in GII.4. Conversely, the capsid diameter of HuNoV GII.7 VLPs appears to expand, alongside a noted decrease in stability, at pH 7.0 and above [88]. 

As enteric viruses, both HuNoV and MNV must withstand significant changes in pH. The collapse of the capsid shell at acidic pH in MNV may be, in part, a protective mechanism to withstand the extremely low pH found in the stomach [80]. Therefore, it would not be surprising if HuNoV adopted a similar mechanism, as evidenced by the epidemic GII.17 Kawasaki strain of HuNoV which demonstrated markedly greater stability at low pH than the non-epidemic GI.1 strain. This may suggest that virulent genotypes have evolved a similar protective mechanism to allow for greater survival in low-pH environments [89]. However, it is unknown if this is due to P-domain collapse into the ‘resting’ conformation. Further research is needed to elucidate the structural impact on HuNoV capsids following changes in pH, and if this same phenomenon can be observed for other *Caliciviridae*. 

## 7. Capsid Priming via Cofactor Interactions

Many caliciviruses engage with cofactors that are not thought to be the primary receptor. TuV, MNV, porcine sapovirus, and FCV are known to interact with sialic acids [90,91,92,93] whereas TuV, HuNoV, and RHDV bind histo-blood group antigens (HBGAs) [94] and bile acids such as GCDCA may be cofactors for TuV, MNV, and some genotypes of HuNoV [90,93,95,96]. 

Research investigating the interaction of MNV with GCDCA revealed an increase in infectivity across several strains of MNV by enhanced binding of the proteinaceous receptor CD300lf [79]. Structural analysis revealed that treatment with GCDCA caused the P-domains to rotate and collapse onto the shell [78]. Further to this, unlike pH treatment, in which the CR6 strain exhibited an intermediate phenotype between ‘resting’ and ‘rising’ [66], GCDCA treatment caused an equivalent level of P-domain contraction across CR6 and the more virulent MNV and WU23 [84]. Therefore, bile acid binding could be considered to ‘prime’ VP1 by inducing a more sterically favourable conformation for receptor interactions. Interestingly, the contraction of P-domains also appears to further increase bile acid affinity, due to the upwards motion of the C’D’ loop that subsequently exposes the bile acid binding pocket [66]. Together, this suggests that, within the metal-rich environment in the gut, both bile acids and metal ions work in synergy to ensure that the greatest population of viruses is in an advantageous conformation for receptor binding and epitope occlusion [67]. 

The non-epidemic HuNoV GI.1 genotype is unable to bind HBGAs in the absence of bile acids [95], whereas upon bile acid binding the P-domain loops were stabilised, providing the basis for HBGA binding through an aspartic acid side chain at position 375 interacting with a fucose moiety on the HBGA surface. Conversely, the epidemic GII.4 and GII.10 genotypes did not require bile acid binding or other cofactor interactions to bind HBGAs. In these cases, the presence of amino acid substitutions in the site of the bile acid binding pocket would likely have occluded bile acid–capsid interactions. These evolutionary adaptations in the P2-domain may work in concert with changes in other regions of VP1 to dramatically decrease bile acid affinity in epidemic genotypes. For example, sequence changes at a loop structure at residues 390 to 395 in HuNoV GII.4 may provide additional interactions for HBGA binding [97], suggesting there may be a reduced necessity for bile acid-mediated capsid priming in epidemic strains. 

An additional explanation for the lack of cofactor requirement in virulent strains may be that viruses in these groups exhibit increased P-domain mobility. Viruses with more mobile P-domains may undergo conformational transitions at a greater rate. Therefore, there may be less dependency on cofactor interactions to ‘prime’ less mobile P-domains into a suitable position. Cryo-EM structures of MNV strains CR6 and WU23 support this; the P-domains of the less virulent CR6 are relatively well-resolved compared to those of the more virulent WU23 [84].

**Figure 4:**
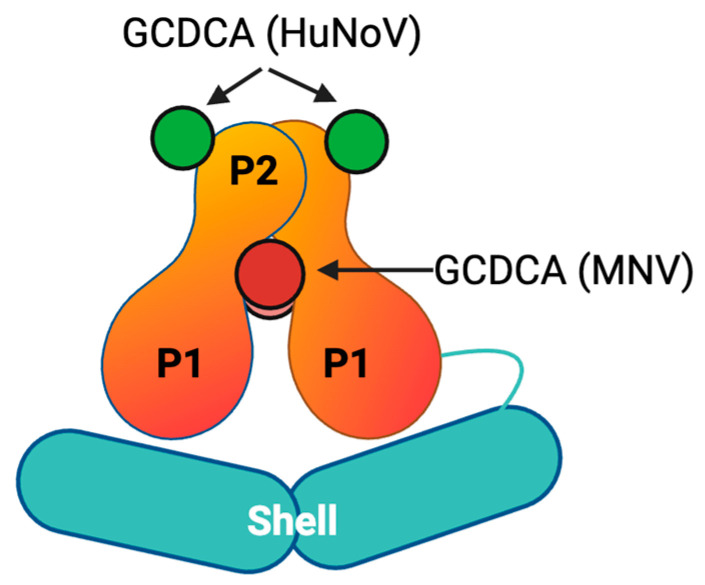
Simplified schematic of GCDCA binding locations on HuNoV (green) and MNV (red) P-domains. Image created in Biorender. GCDCA binds MNV in a hydrophobic binding pocket, with each P-domain binding two bile acid molecules [79]. Conversely, GCDCA appears to bind HuNoV in a bile acid binding pocket at the apical sides of P-domains, being held by four to six residues (depending on strain) with a network of hydrophobic interactions and each P-domain binding a single bile acid molecule.

An interesting note of comparison between HuNoV and MNV bile acid binding lies in the role of GCDCA as a stabilizing factor for MNV P-domains, whereas this does not appear to be necessary for HuNoV. While P-domains are often depicted as dimers, they have been identified as existing in monomeric forms in both MNV and HuNoV [79,98]. Subsequently, Creutznacher et al. demonstrated that incubation with GCDCA significantly stabilized MNV P-domains in a dimeric form; other, weakly binding bile acids such as taurochenodeoxycholic acid (TCDCA) did not appear to have a similar effect [99]. The GCDCA binding site appeared to be located at the interface between the P-dimers, therefore stabilizing them at a central location [79,99] (Figure 4). This model is not dissimilar to the stabilization of P-dimers at central positions via a cadmium ion in HuNoV [69] and a Cys^452^-Cys^452^ disulphide bond in TuV [71]. This stabilization of P-dimers could contribute to successful interaction of MNV with CD300lf [79]. This is further supported by chemical shift perturbation (CSP) experiments, which suggest that binding of GCDCA caused conformational changes at areas remote to the GCDCA binding site, such as at the CD300lf binding loop [99]. 

Conversely, GCDCA exhibits a greatly reduced affinity to HuNoV P-dimers. This might be due to the significantly slower dissociation rate of HuNoV P-domain dimers into monomers; approximately six orders of magnitude smaller than for MNV, consistent with stronger HuNoV P-domain dimer interactions not requiring additional stabilizing cofactors. However, HuNoV bile acid binding sites have been identified as partially conserved pockets at the apical sides of the P-domains, rather than at the dimer interface [95,100]. Binding of GCDCA to these sites appears to stabilize the HBGA binding loop and allows HBGA binding to occur [95]. 

Therefore, despite binding to distinct sites on MNV and HuNoV capsids, GCDCA appears to play a similar role in promoting receptor and/or cofactor interaction in both cases via stabilization of different regions of the P-domains. This activity highlights how, despite strong structural similarities between P-domains of different viruses and binding of similar ligands, the interactions between caliciviruses and target cells are regulated through diverse mechanisms. 

The coxsackie and adenovirus receptor (CAR) is an essential receptor for all recoviruses, including TuV [101], yet it has also been established that HBGA binding enhances the infectivity of TuV, which has been suggested to be a result of binding-induced conformational activation [102]. However, further structural studies are required to confirm this hypothesis. The GI.1 strain of TuV binds both HBGAs and sialic acids for infection of CHO cells; however, this does not appear to be the case for all recoviruses. In contrast, the GI.2 recovirus strain FT285 cannot utilise sialic acid and the GI.3 recovirus FT7 does not require HBGA binding for infection [103], highlighting interesting, and understudied, aspects of strain-specific differences in cofactor binding across the Recovirus genus.

## 8. Post-Translational Modifications

While this review has discussed the role of metal ions, pH, and cofactor receptors in modulating P-domain conformational changes, recent research has found that post-translational modifications (PTMs) may also affect P-domain flexibility.

A spontaneous post-translational modification of asparagine 373 (N373), located in the antigenic loop adjacent to the HuNoV HGBA binding site, into an iso-aspartate residue (isoD373) was previously identified in HuNoV strains GII.4 and MI0011 [104]. The deamidation of this residue meant that HBGA ligands could only interact with isoD373 via one hydrogen bond, rather than the usual two hydrogen bonds when in the N373 form. Therefore, this PTM resulted in the dramatic loss of HBGA affinity. Additionally, P2-domains with the deamidated residues appeared to exhibit significantly greater flexibility than those without.

HuNoV strains lacking an asparagine residue at this position did not appear to undergo deamidation, suggesting this PTM is highly specific. It is interesting, therefore, that the majority of GII.4 strains appear to have an asparagine at this site. As this PTM appeared to spontaneously occur after 1–2 days at pH 7.3 and 37 °C, it may play a role in evading antibody recognition by altering P2-domain conformational dynamics and therefore epitope exposure over the course of HuNoV infection. Interestingly, populations of GII.4 with partially deamidated P-domains appear still able to interact with glycans [98]. Alternatively, it has been suggested that the increased flexibility caused by deamidation may allow for successful host cell attachment and subsequent binding to the as-yet-unknown HuNoV receptor, with glycan interaction still possible in a partially deamidated state. It is interesting to consider, as Dülfer et al. mention, if the P-domain structural flexibility caused by glycan binding could propagate throughout the virus particle to the S-domains, causing sufficient particle destabilisation for uncoating.

It has also been suggested that several sites in the HuNoV P-domains may be susceptible to glycosylation, specifically O-GlcNAcylation. In a cellular context, this modification is associated with essential cellular functions such as transcription, translation, and innate immunity [105,106]. Additionally, glycosylation of viruses such as Zika virus, influenza, and SARS-CoV-2 has been suggested to promote viral binding to cells and epitope occlusion [107,108,109].

Expression of recombinant GII.4 VLPs using a baculovirus vector identified several glycosylation sites on the HuNoV capsid [110]. Multiple residues located in the receptor-binding P2-domain ((Thr350, Thr369, Thr371, Thr381) as well as in the P1-domain (Thr238, Ser519) were identified as modified using mass spectromic analysis. As some of these residues—Thr369, Thr371, and Thr381—are located near the aforementioned N373-containing antigenic loop, modification at these sites may alter glycan binding susceptibility or conformational stability. Additionally, the presence of these modifications might contribute to the occlusion of otherwise exposed epitopes, particularly on the P2-domain. However, the likelihood of these modifications occurring in vivo cannot be extrapolated from these data, as the model used is a modified VLP system in the context of insect cells.

Ultimately, post-translational modifications represent an interesting and understudied area of research that could offer further insight into the modulation of P-domain dynamics.

## 9. P-Domain Mobility Poses a Reproducibility Challenge

**Figure 5:**
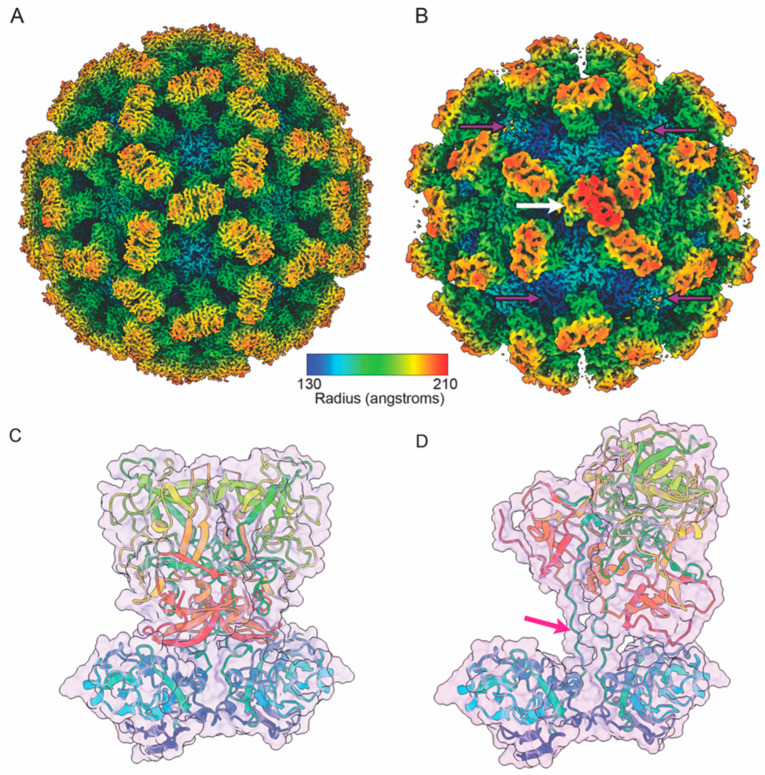
Cryo-EM maps of different preparations of TuV show either (**A**) well-defined, resting pose P-domains, or (**B**) poorly resolved C/C-dimers (purple arrows), that are shown to be in a raised and tilted pose by focussed classification (white arrow). Atomic models of C/C-dimers in resting (**C**) and raised (**D**) states show how the P-dimer tilts and rotates, by unfolding of the S-P linker at amino acid residues Pro203-Pro211 (pink arrow).

Conformational rearrangements of the capsid spikes, revealed by cryo-EM of purified virions and virus-like particles, have led to the publication of conflicting data in cases where the environmental cues required to induce these changes were yet to be defined. This can be perplexing and poses a reproducibility challenge to those researchers investigating virion structure and function. Indeed, in our own hands we have found that both FCV and TuV can vary between preparations—sometimes showing well-defined resting pose P-dimers and sometimes showing poorly defined capsid spikes that are then revealed to be in different poses across the data set, using symmetry-breaking reconstruction methods. For FCV we have revised our purification protocols such that we can produce consistent preparations. For TuV, however, we are yet to discern conditions for reproducibly preparing virions with resting-state P-domains. We have produced a 2.6 angstrom resolution cryo-EM reconstruction from one preparation in which the P-domains were well-resolved in a resting state. In subsequent preparations we determined cryo-EM maps at 3 angstrom net resolution in which C/C-dimers were poorly resolved. Focused classification confirmed conformational flexibility at this site showing that the P-dimers were rotated and tilted away from the icosahedral two-fold axes, breaking symmetry and resulting in incoherently averaged density for this region (Figure 5 and Supplemental movie). In these preparations, the addition of metal ions, known entry cofactors GCDCA, HBGA, or the receptor coxsackie-adenovirus receptor (CAR) were not able to stabilize the C/C dimer. In the absence of a clearly defined environmental cue that triggers these conformational changes, it is to be expected that neutralization and entry studies may yield inconsistent data when using different preparations of virus.

## 10. The Role of P-Domain Rotation in Virus Entry

Alongside their putative role in immune evasion and receptor engagement, the flexible nature of the P-domains plays a critical role in initiating calicivirus infection. For FCV, we have shown that, following engagement with the fJAM-A receptor, VP1 undergoes significant structural rearrangement. A loop consisting of residues 436–448 on the tip of the P2-domain rises upwards to meet fJAM, forming multiple hydrogen bonds. This movement leads to the formation of a cleft in the P2-domain, which is not present in the undecorated virion. The opening of this cleft allows for 12 copies of the minor capsid protein VP2 to assemble and form a large portal assembly [22]. We hypothesised that the portal forms a ‘channel’ through which the viral genome is delivered into the host cell cytoplasm to initiate infection. Recent work by Sun et al. supports this hypothesis; the hydrophobic N-terminal residues of VP2 appear to pierce the membranes of early endosomes, allowing for viral gRNA release in moderately acidic (pH 6.2) conditions [23]. Alanine substitution of highly conserved VP2 residues (aa 4–11) significantly decreased the rate of gRNA release and negatively impacted plaque size and replication titre, highlighting the requirement for VP2 portal formation prior to successful infection.

Although formation of the VP2 portal has only been identified to date in FCV strains F9 and HRB-SS, these data explain why deletion of VP2 resulted in non-infectious viral particles in studies of FCV Urbana [9], MNV [111], and TuV [112]. Interestingly, VP2 deletion does not appear to have any effect on RHDV infectivity, suggesting that this mechanism might not be fully conserved across the *Caliciviridae* [113].

To our knowledge, the effect of VP2 deletion on HuNoV infectivity has not yet been determined. Nevertheless, it is evident that VP2 plays a critical and, as yet, understudied, role in infectivity for most caliciviruses; and P-domain conformational changes are likely essential to enable VP2 portal assembly and thereby facilitate virus entry.

## 11. Discussion and Future Perspectives

The ability of P-domains to undergo conformational shifts according to environmental conditions plays a crucial role in infection, significantly influencing both receptor binding and entry, and enabling evasion of the host immune response. This conformational flexibility is well-conserved across the *Caliciviridae*.

While these discoveries represent important steps forward in our understanding of calicivirus infection, several broader questions remain. A significant proportion of work in this area has been conducted using MNV, as well as (to a lesser extent) FCV and certain HuNoV genotypes in the form of VLPs. While it can be assumed that the mechanism and purpose of P-domain movement is well-conserved among the *Caliciviridae*, it is still important to characterise P-domain conformational dynamics in other HuNoV surrogates, such as TuV and porcine sapovirus, as well as enteric strains of FCV [77]. Indeed, the extent to which this mechanism is conserved is an interesting question to consider even among the more well-characterised *Caliciviridae*; an increased understanding of P-domain mobility could provide the foundations for improved rational vaccine design, which would benefit both human and veterinary medicine.

Much of the work described here has been performed using norovirus surrogates or VLPs, due to the limited cultivability of HuNoV in cell culture. However, it should be noted that several advances have been made in this area that might, in the future, allow for the study of P-domain movement in authentic HuNoV.

Successful replication of multiple GII genotypes was observed in stem-cell-derived human intestinal enteroids (HIEs), as well as B cells [114,115]. The addition of known HuNoV cofactors such as bile acids has also been shown to promote replication of otherwise uncultivable genotypes [116], and further work has identified consistent replication of multiple genotypes in J2 HIE via a combination of GCDCA supplementation together with specialised growth and differentiation media [117]. Furthermore, genetic modification of cell lines to promote fucosylation of HBGAs [118] and investigation into the role of HIE innate immune responses in restricting HuNoV replication [119] continue to provide insight into the optimisation of cell lines for the culture of HuNoV. Continued improvements in this area will contribute to the establishment of consistent, reproducible, and high-titre in vitro HuNoV propagation systems.

It has been noted previously that C/C dimers exhibit a greater degree of flexibility than A/B dimers in TuV, FCV, and RHDV [39,64,120]. In TuV and RHDV, this increased flexibility appeared to be caused by significantly reduced interactions between the shell and P1-domain in C/C dimers, compared to A/B dimers.

While the purpose of this is unknown, it was suggested that this particularly flexible state could facilitate interactions with host cell co-factors. However, this has not been observed in MNV, with MNV A/B dimers appearing to exhibit greater flexibility than MNV C/C dimers in some cryo-EM preparations [121]. This might be due to differing S-P1 interactions between different viruses; alternatively, these preparations could have been affected by reproducibility issues when attempting to resolve C/C dimers.

Nevertheless, the effects of different conformational dynamics between A/B and C/C dimers should be taken into account when considering calicivirus interactions with receptors and cofactors.

P-domains pose an attractive target for potential antivirals. A small selection of nanobody-based inhibitors targeting the HuNoV HBGA binding site have shown some success in blocking virus-HBGA interactions [122]. However, some nanobodies might require the P-domain to be in the ‘rising’ conformation in order to bind fully, thereby limiting their effectiveness. Also, since the HBGA binding site varies significantly between genotypes, antivirals might be selective towards only a small subset of viruses. Alternative strategies could involve ‘locking’ the P-domain in either the resting or rising conformation, for example using small-molecule or peptide-based inhibitors; the complete neutralisation of HuNoV GII.4 was demonstrated recently using a nanobody that targeted a region on the P-domains that is only exposed in the ‘raised‘ conformation, demonstrating the possibility of developing novel therapeutics that exploit the intrinsic flexibility of this important region [123].

## Data Availability

Data for the resting state Tulane virus structure were deposited in public repositories: raw data micrograph movies (https://www.ebi.ac.uk/empiar/) EMPIAR-12434, cryo-EM map (https://www.ebi.ac.uk/emdb/) EMDB-52037, atomic model (https://www.ebi.ac.uk/pdbe/) PDB-9HC5. As the raised-state model is pseudoatomic (owing to insufficient resolution), the data are not deposited at this time but will be provided on request by the senior author.

## Supplementary Materials

The following supporting information can be downloaded at: https://www.mdpi.com/article/10.3390/v16121835/s1, Table S1: Table to show strain details and Genbank accession numbers for VP1 sequences used for preparation of Figure 2.; Video S1: Movies to show a morph between conformations resolved at the CC dimer between preparations of Tulane virus.
